# Momentary Loneliness Among Older Adults: Real-Time Correlates and Potential Entry Points for Intervention

**DOI:** 10.1093/geroni/igaf122.462

**Published:** 2025-12-31

**Authors:** Nell Compernolle, Louise Hawkley, Ellen Bloss

**Affiliations:** NORC at the University of Chicago, Chicago, Illinois, United States; National Opinion Research Center at The University of Chicago, Chicago, Illinois, United States; NORC at the University of Chicago, Chicago, Illinois, United States

## Abstract

**Background and Objectives:**

Momentary loneliness remains at the forefront of investigations into the complexities of loneliness. Ecological momentary assessments (EMAs) have enabled researchers to consider the relevance of real-time social context for loneliness rather than rely on the long-documented more stable measures of social integration. Here, we summarize a series of studies that provide novel insights into who may be more likely to experience momentary loneliness, when, and implications for intervention.

**Methods:**

We use data from the Chicago Health and Activity Space in Real Time study (CHART), a population-based cluster sample of 450 adults ages 65+. A series of regression models examined associations between real-time physical and social context and momentary loneliness, as well as moderating effects of more stable social factors and socio-demographic characteristics. Subsequent focus group discussions explored the nuance to older adults’ momentary loneliness experiences.

**Results:**

Findings identify being momentarily alone and/or at home as key risk factors for momentary loneliness. However, these loneliness-inducing contexts were stronger for certain sub-populations, those with large personal networks, and during the COVID-19 pandemic (versus before). Qualitative research highlights social company departures, misalignment in socializing desires, and a lack of belonging as additional real-time factors increasing risk.

**Discussion:**

Results are among the first to identify contextual effects on real-time loneliness in older adults and how these associations vary across individuals and more stable and societal-level contexts. Group discussions further inform the feasibility and utility of a just-in-time adaptive intervention to reduce momentary loneliness via key antecedents personalized to users.

